# Case of Vicarious Menstruation

**Published:** 1882-07

**Authors:** 


### CASE OF VICARIOUS MENSTRUATION.

Mr. Stear reported, at the meeting of the Cambridge Medical Society, held April 11th, a case of vicarious menstruation from the nipples, occurring in a healthy woman aged fifty, who had been married many years, but had never been pregnant. She stated that menstruation commenced at the age of thirteen, and had been regular and normal until about two years ago, when it ceased. For twelve months past, however, she had suffered from a discharge of blood from the nipples, which recurred every month and lasted from three to four days, the quantity of blood being such that she was obliged to wear a napkin. The breasts at these times were very painful, the pain being similar in character to that which she had always experienced when menstruating normally. The mammæ were large, but presented no abnormal appearance. There could be no doubt as to the genuineness of the case, as he had himself seen her more than once when the discharge was present; moreover, his patient had been much alarmed by its occurrence, and showed great anxiety to be relieved. Professor Paget said that many years ago he had seen a young girl at the Moorfields Hospital who every month had a small effusion of blood into the anterior chamber of the eye at the menstrual period, the effusion becoming absorbed during the intervals.—*Lancet,* May 13, 1882.

